
JOURNAL INFORMATION
==============================
NLM Title Abbreviation: ScientificWorldJournal
Journal ID: ScientificWorldJournal
NLM Journal ID: 101131163
Journal ID: TSWJ

The Scientific World Journal

ISSN: 2356-6140
EISSN: 1537-744X
Publisher: Wiley

ARTICLE INFORMATION
==============================
PMCID: PMC6084202
PMID: 30147483
DOI: 10.1100/tsw.2001.145
Article ID: 402065
Article version: 1
Subjects: Short Report

Regulation of T-Cell Apoptosis by Sequences Encoded at the Luca-15 Candidate Tumour Suppressor Locus

Mourtada-Maarabouni Mirna 1 *
Sutherland Leslie C. 1
Clark Jeremy P. 2
Cooper Colin S. 2
Williams Gwyn T. 1
1 School of Life Sciences, Keele University, Staffordshire, U.K. ST5 5BG, UK
2 Molecular Carcinogenesis, Haddow Labs, Institute of Cancer Research, Sutton, SMG2 5NG, UK
*Mirna Mourtada-Maarabouni: m.mourtada@biol.keele.ac.uk
Publication date: 2001
Electronic publication date: 2001 Nov 15
Volume: 1
First page: 38
Last page: 38
Copyright: Copyright © 2001 Mirna Mourtada-Maarabouni et al.
Copyright year: 2001
License: This is an open access article distributed under the Creative Commons Attribution License, which permits unrestricted use, distribution, and reproduction in any medium, provided the original work is properly cited.
License URL: https://creativecommons.org/licenses/by/3.0/

Funding: Wellcome Trust

==============================
Miami Nature Biotechnology Short Reports
TheScientificWorld (2001) 1(S3), 40SR
ISSN 1532-2246; DOI 10.1100/tsw.2001.145

### REGULATION OF T-CELL APOPTOSIS BY SEQUENCES ENCODED AT THE LUCA-15 CANDIDATE TUMOUR SUPPRESSOR LOCUS

Mirna Mourtada-Maarabouni*(1), Leslie C. Sutherland (1), Jeremy P. Clark (2), Colin S. Cooper (2) and Gwyn T. Williams (1)

(1)School of Life Sciences, Keele University, Staffordshire, U.K. ST5 5BG. (2)Molecular Carcinogenesis, Haddow Labs, Institute of Cancer Research, Sutton, SMG2 5NG
* m.mourtada@biol.keele.ac.uk

**INTRODUCTION**. Using an episomal retrieval functional expression cloning system(1), a 431 bp fragment of cDNA (je2 ) was identified, which strongly inhibited Fas-mediated apoptosis in Jurkat human T-cells(2). Je2 maps to the LUCA22 human locus at 3p21.3, a highly significant lung cancer tumour suppressor locus (2), and lies in the antisense orientation within an intron of Luca-15 gene. We have investigated the effect of Je2 on apoptosis in another human T-cell line CEM-C7 and using DNA microarrays, we report the alteration of the expression of several genes by Je2.

**METHODS**. Je2 was subcloned into pcDNA3 and was introduced into CEM-C7 cells by electroporation. Stable clones were exposed to 5ng/ml anti-Fas for 24h, after that time, cell viability was determined by vital dye staining. Details for the sample preparation and microarray processing will be described elsewhere. Fold changes in gene expression were determined by using GenePix 2 software.

**RESULTS.** Stable expression of the antisense je2 sequence produced marked inhibition of Fas-mediated apoptosis (Fig.1). Je2 transfected clones maintained the ability to proliferate in the continuous presence of anti-Fas, and were also protected from apoptosis induced by other stimuli (not shown).

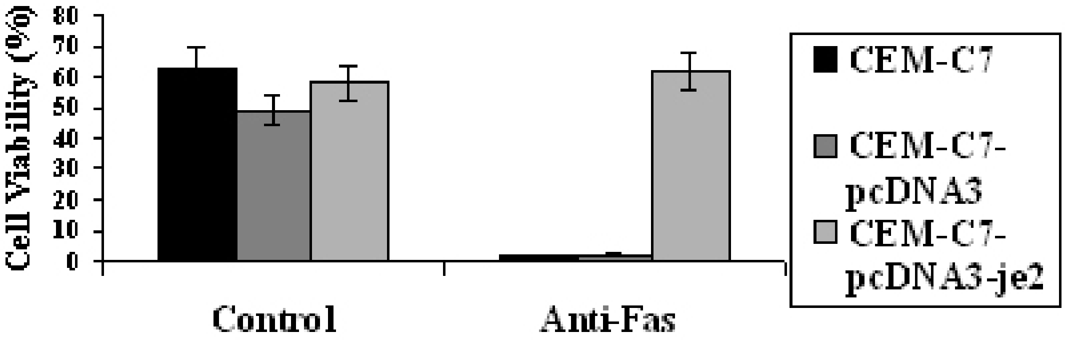

In addition, stable expression of je2 altered the expression of several genes, some of these genes were previously reported to regulate apoptosis. Some of the genes that were differentially expressed are listed in Table 1.

| ID | Fold | Entrez definition |
|---|---|---|
| Genes down-regulated | | |
| 241481 | 5 | Caspase-10 |
| 724831 | 6 | B-cell lymphoma protein 7B |
| 509641 | 15 | Interferon- inducible protein |
| 712840 | 17 | Transcription factor Stat5b |
| Genes up-regulated | | |
| 213136 | 12 | B-cell translocation gene 2(BTG2) |
| 813630 | 7 | Pim-1-oncogene |
| 221846 | 5 | Checkpoint suppressor 1- mRNA |

**DISCUSSION.** Sequence homology searches revealed that je2 maps to 3p21.3, to an intronic region of LUCA-15. Northern blotting studies have revealed clear evidence for alternative splicing of the LUCA-15 gene transcripts (2). Over-expression of a splice variant of LUCA-15, clone 26, is reported to inhibit cell proliferation and sensitise the cells to Fas-mediated apoptosis (2). It is therefore attractive to speculate that like other apoptosis regulators, different transcripts of LUCA-15 together encode both inducers and suppressors of apoptosis. Our observation suggests that je2 function to suppress cell death and indicates that LUCA-15 can regulate the core mechanism of apoptosis.

**ACKNOWLEDGEMENT.** This work was supported by the Wellcome Trust.

**REFERENCES.**

1. Yates, J., Warren, N., and Sudgen, B. (1985) Nature 313, 812-815
2. Sutherland, L.C., Gill, S.E., Cable, H.C., Poirier, G.G., Miller, B.A., Cooper, C.S., and Williams, G.T. (2000) Oncogenes 35(19), 3774-3778
